# (*E*)-5-(4-Chloro­benzyl­idene)-1-phenyl-4,5,6,7-tetra­hydro-1*H*-indazol-4-one: crystal structure and Hirshfeld surface analysis

**DOI:** 10.1107/S2414314621011950

**Published:** 2021-11-16

**Authors:** C. Selva Meenatchi, S. Athimoolam, J. Suresh, S. Raja Rubina, R. Ranjith Kumar, S. R. Bhandari

**Affiliations:** a Department of Physics, The Madura College, Madurai 625 011, India; b Department of Physics, University College of Engineering Nagercoil, Anna University, Nagercoil 629 004, Tamilnadu, India; c Department of Organic Chemistry, School of Chemistry, Madurai Kamaraj University, Madurai 625 021, India; d Department of Physics, Bhairahawa M. Campus, Tribhuvan University, Nepal; Sunway University, Malaysia

**Keywords:** crystal structure, indazol-4-one, Hirshfeld Surface

## Abstract

In the title compound, C_20_H_15_ClN_2_O, the non-aromatic six-membered ring adopts a distorted envelope conformation with methyl­ene-C atom nearest to the five-membered ring being the flap atom. The dihedral angle between the phenyl and chloro­benzene rings is 74.5 (1)°. The heterocyclic ring forms dihedral angles of 37.9 (1) and 64.3 (1)° with the phenyl and chloro­benzene rings, respectively. In the crystal, weak C—H⋯O inter­actions feature predominantly within the three-dimensional architecture. The inter­molecular inter­actions are further analysed with the calculation of the Hirshfeld surfaces highlighting the prominent role of C—H⋯O inter­actions, along with H⋯H (36.8%) and C⋯H/H⋯C (26.5%) contacts.

## Abstract 2

The title 1,2-diazole derivative is highly twisted with the dihedral angle between the pendant rings being 74.5 (1)°. In the crystal, weak C—H⋯O inter­actions feature predominantly within the three-dimensional architecture.

## Graphical abstract

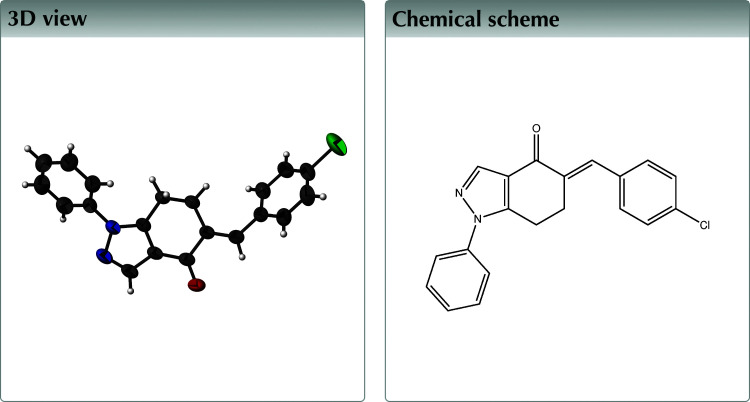

## Structure description

Many heterocyclic compounds are studied for their biological and pharmacological activities. For example, 1,2-diazole derivatives are known to possess anti-depressant, anti-viral, anti-inflammatory and anti-cancer activities (Popat *et al.*, 2003 ▸; Faisal *et al.*, 2019 ▸). The crystal and mol­ecular structure of one such indazole derivative, namely, (*E*)-5-(4-chloro­benzyl­idene)-1-phenyl-4,5,6,7-tetra­hydro-1*H*- indazol-4-one, is reported herein.

**Figure 1:**
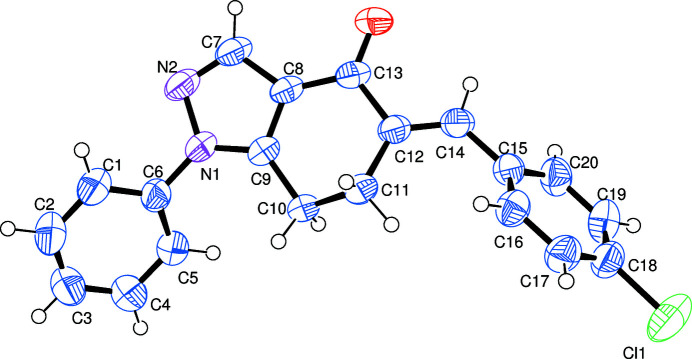
The mol­ecular structure of the title compound, showing 50% probability displacement ellipsoids

The non-aromatic six-membered ring adopts a distorted envelope conformation with the methyl­ene-C10 atom being the flap atom, Fig. 1 ▸. The heterocyclic ring forms dihedral angles of 37.9 (1) and 64.3 (1)° with the phenyl and chloro­benzene rings, respectively. The dihedral angle between the pendant rings is 74.5 (1)°. The mol­ecular structure features a weak intra­molecular inter­action through C14—H14⋯O1 (Table 1 ▸).

**Figure 2:**
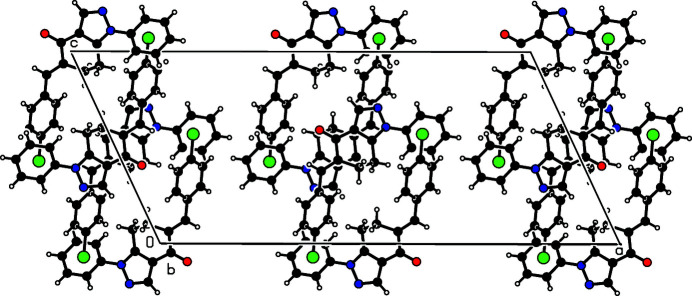
A view of the unit-cell contents viewed in projection down the *b*-axis.

**Figure 3:**
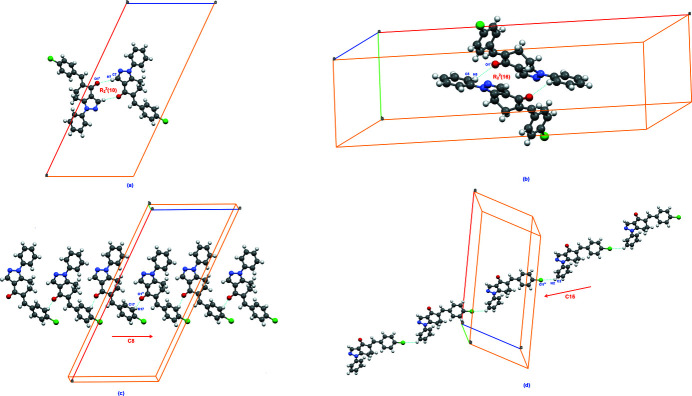
Views of significant C—H⋯*X* inter­actions (*X* = O or Cl) shown as dashed lines forming (*a*) an 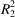 (10) ring motif, (*b*) an 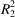 (16) ring, (*c*) a *C*(8) chain motif and (*d*) a *C*(15) chain.

The mol­ecular packing features two ring motifs, *viz*., 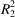 (10) and 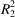 (16) (Bernstein *et al.*, 1995 ▸), each around an inversion centre, through two C—H⋯O inter­actions, *i.e*. C7—H7⋯O1^ii^ and C5—H5⋯O1^i^, respectively, Fig. 2 ▸; for symmetry codes, refer to Table 1 ▸. The centrosymmetric dimers thus formed are connected through two C—H⋯*X* inter­actions, *viz*., C17—H17⋯O^iii^ and C2—H2⋯Cl^iv^, leading to chain *C*(8) and *C*(15) motifs, respectively. The first named inter­action serves to connect the mol­ecules along the along [001] and the latter along [101], Fig. 3 ▸. Clearly, the carbonyl-O1 atom plays a pivotal role in the supra­molecular assembly.

**Figure 4:**
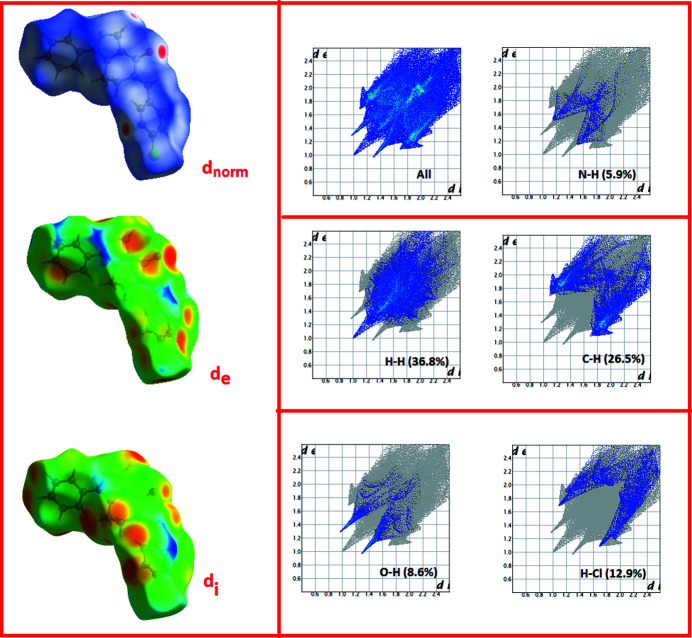
Hirshfeld three-dimensional surfaces (showing *d*_norm_, *d*_i_ and *d*_e_) and selected two-dimensional fingerprint plots

The inter­molecular inter­actions in the crystal state can be visualized through the calculation of the Hirshfeld surfaces and associated two-dimensional fingerprint plots. These were generated by *Crystal Explorer* (Wolff *et al.*, 2012 ▸). The Hirshfeld surface is colour-mapped with the normalized contact distance, *d*_norm_, *i.e*. from red (distances shorter than the sum of the van der Waals radii) through white to blue (distances longer than the sum of the van der Waals radii). The different types of inter­molecular inter­actions can be identified by colour-coding distances from the surface to the nearest atom exterior (*d*_e_) or inter­ior (*d*_i_) plots to the surface. The three-dimensional Hirshfeld surfaces and selected two-dimensional fingerprint plots (with percentage contributions) are given in Fig. 4 ▸.

The presence of spikes due to O⋯H/H⋯O inter­actions (8.6%) correspond to C—H⋯O inter­molecular inter­actions, which feature predominantly within the crystalline assembly. The contribution of C⋯H/H⋯C contacts (26.5%), leading to a pair of well-defined wings, is also noteworthy. The H⋯H inter­actions contribute 36.8% with widely scattered points of high density, which is consistent with the large number of hydrogen atoms at the surface of the mol­ecule. The Cl⋯H/H⋯Cl contacts also make a notable contribution to the total Hirshfeld surfaces, comprising about 12.9%. The large number of H⋯H, Cl⋯H/H⋯Cl, O⋯H/H⋯O inter­actions suggest that van der Waals inter­actions play a significant role in the packing in the crystal.

## Synthesis and crystallization

A mixture of 1-phenyl-1,5,6,7-tetra­hydro-4*H*-indazol-4-one, (1 mmol) and 4-chloro­benzaldehyde (1 mmol) was dissolved in ethanol followed by the addition of NaOH. The resulting mixture was stirred at room temperature for 1 h to afford (*E*)-5-(4-chloro­benzyl­idene)-1-phenyl-1,5,6,7-tetra­hydro-4*H*-ind­a­zol-4-ones as the precipitate. This was filtered off and recrystallized from ethanol to afford colourless crystals; yield: 95%, m.p. 183–184°C.

## Refinement

Crystal data, data collection and structure refinement details are summarized in Table 2 ▸.

## Supplementary Material

Crystal structure: contains datablock(s) I, global. DOI: 10.1107/S2414314621011950/tk4071sup1.cif

Structure factors: contains datablock(s) I. DOI: 10.1107/S2414314621011950/tk4071Isup2.hkl

Supporting information file. DOI: 10.1107/S2414314621011950/tk4071Isup3.cml

CCDC reference: 2121290

Additional supporting information:  crystallographic information; 3D view; checkCIF report

## Figures and Tables

**Table 1:** Hydrogen-bond geometry (Å, °)

*D*—H⋯*A*	*D*—H	H⋯*A*	*D*⋯*A*	*D*—H⋯*A*
C14—H14⋯O1	0.93	2.43	2.804 (3)	104
C5—H5⋯O1^i^	0.93	2.53	3.320 (3)	143
C7—H7⋯O1^ii^	0.93	2.59	3.493 (3)	163
C17—H17⋯O1^iii^	0.93	2.40	3.260 (3)	154
C2—H2⋯Cl1^iv^	0.93	2.90	3.633 (3)	137

Symmetry codes: (i) 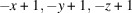 ; (ii) 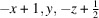 ; (iii) 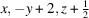 ; (iv) 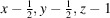 .

**Table 2:** Experimental details

Crystal data	
Chemical formula	C_20_H_15_ClN_2_O
*M*_r_	334.79
Crystal system, space group	Monoclinic, *C*2/*c*
Temperature (K)	293
*a*, *b*, *c* (Å)	30.4808 (16), 8.6604 (5), 14.0457 (7)
β (°)	115.071 (2)
*V* (Å^3^)	3358.4 (3)
*Z*	8
Radiation type	Mo *K*α
μ (mm^−1^)	0.24
Crystal size (mm)	0.22 × 0.20 × 0.16

Data collection	
Diffractometer	Bruker *SMART* APEXII CCD
Absorption correction	–
No. of measured, independent and observed [*I* > 2σ(*I*)] reflections	42189, 2949, 2353
*R*_int_	0.056
(sin θ/λ)_max_ (Å^−1^)	0.606

Refinement	
*R*[*F* ^2^ > 2σ(*F* ^2^)], *wR*(*F* ^2^), *S*	0.051, 0.146, 1.12
No. of reflections	2949
No. of parameters	218
H-atom treatment	H-atom parameters constrained
Δρ_max_, Δρ_min_ (e Å^−3^)	0.39, −0.44

Computer programs: *APEX2* and *SAINT* (Bruker, 2009 ▸), *SHELXT* (Sheldrick, 2015*a* ▸), *SHELXL97* and *SHELXL2018* (Sheldrick, 2015*b* ▸), *ORTEP-3 for Windows* (Farrugia, 2012 ▸), *Mercury* (Macrae *et al.*, 2020 ▸) and *PLATON* (Spek, 2020 ▸).

## full crystallographic data

### Crystal data

**Table:**

C_20_H_15_ClN_2_O	*F*(000) = 1392
*M**_r_* = 334.79	*D*_x_ = 1.324 Mg m^−^^3^
Monoclinic, *C*2/*c*	Mo *K*α radiation, λ = 0.71073 Å
*a* = 30.4808 (16) Å	Cell parameters from 2246 reflections
*b* = 8.6604 (5) Å	θ = 2.9–23.5°
*c* = 14.0457 (7) Å	µ = 0.24 mm^−^^1^
β = 115.071 (2)°	*T* = 293 K
*V* = 3358.4 (3) Å^3^	Block, colourless
*Z* = 8	0.22 × 0.20 × 0.16 mm

### Data collection

**Table:**

Bruker SMART APEXII CCD diffractometer	*R*_int_ = 0.056
Radiation source: fine-focus sealed tube	θ_max_ = 25.5°, θ_min_ = 3.2°
ω and φ scans	*h* = −36→36
42189 measured reflections	*k* = −10→10
2949 independent reflections	*l* = −16→16
2353 reflections with *I* > 2σ(*I*)	

### Refinement

**Table:**

Refinement on *F*^2^	Hydrogen site location: inferred from neighbouring sites
Least-squares matrix: full	H-atom parameters constrained
*R*[*F*^2^ > 2σ(*F*^2^)] = 0.051	*w* = 1/[σ^2^(*F*_o_^2^) + (0.0578*P*)^2^ + 3.7997*P*] where *P* = (*F*_o_^2^ + 2*F*_c_^2^)/3
*wR*(*F*^2^) = 0.146	(Δ/σ)_max_ < 0.001
*S* = 1.12	Δρ_max_ = 0.39 e Å^−^^3^
2949 reflections	Δρ_min_ = −0.44 e Å^−^^3^
218 parameters	Extinction correction: SHELXL2018 (Sheldrick, 2015b), Fc^*^=kFc[1+0.001xFc^2^λ^3^/sin(2θ)]^-1/4^
0 restraints	Extinction coefficient: 0.0169 (15)

### Special details

**Table:**

Geometry. All esds (except the esd in the dihedral angle between two l.s. planes) are estimated using the full covariance matrix. The cell esds are taken into account individually in the estimation of esds in distances, angles and torsion angles; correlations between esds in cell parameters are only used when they are defined by crystal symmetry. An approximate (isotropic) treatment of cell esds is used for estimating esds involving l.s. planes.
Refinement. The hydrogen atoms were included in their geometrically calculated positions and refined isotropically with C—H = 0.93 or 0.97 Å and *U*_iso_(H) = 1.2*U*_eq_(C).

### Fractional atomic coordinates and isotropic or equivalent isotropic displacement parameters (Å^2^)

**Table:**

	*x*	*y*	*z*	*U*_iso_*/*U*_eq_	
C1	0.30506 (10)	0.5737 (4)	0.3310 (2)	0.0788 (9)	
H1	0.3008	0.6269	0.2702	0.095*	
C2	0.26559 (11)	0.5226 (5)	0.3465 (3)	0.0966 (12)	
H2	0.2345	0.5414	0.2953	0.116*	
C3	0.27141 (10)	0.4449 (4)	0.4359 (3)	0.0853 (9)	
H3	0.2445	0.4113	0.4450	0.102*	
C4	0.31708 (10)	0.4167 (3)	0.5118 (2)	0.0706 (7)	
H4	0.3212	0.3647	0.5729	0.085*	
C5	0.35712 (9)	0.4656 (3)	0.49759 (19)	0.0580 (6)	
H5	0.3881	0.4453	0.5487	0.070*	
C6	0.35095 (8)	0.5440 (3)	0.40771 (17)	0.0531 (6)	
C7	0.43290 (9)	0.6267 (3)	0.30115 (16)	0.0558 (6)	
H7	0.4422	0.6301	0.2461	0.067*	
C8	0.46319 (8)	0.6698 (3)	0.40535 (15)	0.0466 (5)	
C9	0.43539 (8)	0.6472 (2)	0.46043 (15)	0.0447 (5)	
C10	0.45229 (8)	0.6841 (3)	0.57428 (15)	0.0490 (5)	
H10A	0.4664	0.5931	0.6165	0.059*	
H10B	0.4252	0.7178	0.5882	0.059*	
C11	0.49020 (8)	0.8128 (3)	0.60228 (17)	0.0536 (6)	
H11A	0.4739	0.9090	0.5720	0.064*	
H11B	0.5055	0.8254	0.6780	0.064*	
C12	0.52910 (8)	0.7832 (3)	0.56429 (16)	0.0460 (5)	
C13	0.51238 (8)	0.7266 (3)	0.45363 (16)	0.0480 (5)	
C14	0.57663 (8)	0.8010 (3)	0.62150 (17)	0.0503 (5)	
H14	0.5962	0.7770	0.5876	0.060*	
C15	0.60225 (8)	0.8534 (3)	0.73111 (17)	0.0483 (5)	
C16	0.58651 (9)	0.9775 (3)	0.77156 (18)	0.0545 (6)	
H16	0.5589	1.0315	0.7278	0.065*	
C17	0.61102 (9)	1.0221 (3)	0.87534 (19)	0.0599 (6)	
H17	0.6002	1.1056	0.9011	0.072*	
C18	0.65138 (9)	0.9423 (3)	0.93968 (19)	0.0617 (7)	
C19	0.66861 (9)	0.8207 (3)	0.9027 (2)	0.0666 (7)	
H19	0.6962	0.7676	0.9473	0.080*	
C20	0.64429 (8)	0.7783 (3)	0.7983 (2)	0.0595 (6)	
H20	0.6563	0.6978	0.7725	0.071*	
N1	0.39190 (7)	0.5928 (2)	0.39106 (13)	0.0502 (5)	
N2	0.39011 (8)	0.5812 (2)	0.29111 (14)	0.0588 (5)	
O1	0.53879 (6)	0.7264 (2)	0.40725 (12)	0.0669 (5)	
Cl1	0.68147 (4)	0.99499 (12)	1.07100 (6)	0.1050 (4)	

### Atomic displacement parameters (Å^2^)

**Table:**

	*U*^11^	*U*^22^	*U*^33^	*U*^12^	*U*^13^	*U*^23^
C1	0.0585 (16)	0.120 (3)	0.0481 (14)	0.0063 (16)	0.0129 (12)	−0.0020 (15)
C2	0.0489 (15)	0.156 (3)	0.0687 (19)	−0.0017 (18)	0.0097 (14)	−0.014 (2)
C3	0.0570 (16)	0.119 (3)	0.082 (2)	−0.0160 (17)	0.0322 (15)	−0.0208 (19)
C4	0.0634 (16)	0.0813 (19)	0.0700 (17)	−0.0088 (14)	0.0310 (14)	−0.0017 (14)
C5	0.0515 (13)	0.0658 (15)	0.0528 (13)	−0.0032 (11)	0.0185 (11)	−0.0002 (11)
C6	0.0450 (12)	0.0638 (14)	0.0472 (12)	−0.0030 (10)	0.0163 (10)	−0.0126 (11)
C7	0.0719 (16)	0.0620 (14)	0.0346 (11)	−0.0053 (12)	0.0235 (11)	−0.0014 (10)
C8	0.0588 (12)	0.0498 (12)	0.0326 (10)	−0.0014 (10)	0.0205 (9)	−0.0006 (9)
C9	0.0512 (12)	0.0463 (12)	0.0342 (10)	0.0015 (9)	0.0159 (9)	−0.0006 (8)
C10	0.0503 (12)	0.0643 (14)	0.0354 (10)	−0.0041 (10)	0.0208 (9)	−0.0065 (10)
C11	0.0565 (13)	0.0652 (14)	0.0418 (11)	−0.0061 (11)	0.0234 (10)	−0.0131 (10)
C12	0.0542 (12)	0.0478 (12)	0.0389 (11)	−0.0037 (10)	0.0225 (9)	−0.0017 (9)
C13	0.0612 (13)	0.0496 (12)	0.0380 (10)	−0.0013 (10)	0.0256 (10)	0.0011 (9)
C14	0.0583 (13)	0.0526 (13)	0.0458 (12)	−0.0079 (10)	0.0278 (10)	−0.0038 (10)
C15	0.0494 (12)	0.0484 (12)	0.0476 (12)	−0.0080 (10)	0.0211 (10)	−0.0035 (9)
C16	0.0570 (13)	0.0497 (13)	0.0512 (13)	−0.0031 (10)	0.0175 (11)	−0.0019 (10)
C17	0.0673 (15)	0.0550 (14)	0.0565 (14)	−0.0070 (12)	0.0253 (12)	−0.0130 (11)
C18	0.0647 (15)	0.0654 (16)	0.0472 (13)	−0.0158 (12)	0.0162 (12)	−0.0066 (11)
C19	0.0528 (13)	0.0643 (16)	0.0637 (16)	−0.0029 (12)	0.0064 (12)	−0.0019 (13)
C20	0.0498 (13)	0.0578 (14)	0.0671 (15)	−0.0046 (11)	0.0211 (12)	−0.0139 (12)
N1	0.0526 (10)	0.0598 (12)	0.0343 (9)	−0.0020 (9)	0.0146 (8)	−0.0049 (8)
N2	0.0689 (13)	0.0686 (13)	0.0332 (9)	−0.0044 (10)	0.0163 (9)	−0.0038 (9)
O1	0.0719 (11)	0.0929 (14)	0.0488 (9)	−0.0152 (10)	0.0382 (9)	−0.0104 (9)
Cl1	0.1194 (8)	0.1189 (8)	0.0501 (4)	−0.0154 (6)	0.0101 (4)	−0.0184 (4)

### Geometric parameters (Å, º)

**Table:**

C1—C6	1.380 (3)	C10—H10B	0.9700
C1—C2	1.383 (4)	C11—C12	1.514 (3)
C1—H1	0.9300	C11—H11A	0.9700
C2—C3	1.367 (5)	C11—H11B	0.9700
C2—H2	0.9300	C12—C14	1.335 (3)
C3—C4	1.370 (4)	C12—C13	1.498 (3)
C3—H3	0.9300	C13—O1	1.232 (2)
C4—C5	1.384 (3)	C14—C15	1.472 (3)
C4—H4	0.9300	C14—H14	0.9300
C5—C6	1.375 (3)	C15—C20	1.389 (3)
C5—H5	0.9300	C15—C16	1.393 (3)
C6—N1	1.428 (3)	C16—C17	1.382 (3)
C7—N2	1.312 (3)	C16—H16	0.9300
C7—C8	1.410 (3)	C17—C18	1.366 (4)
C7—H7	0.9300	C17—H17	0.9300
C8—C9	1.382 (3)	C18—C19	1.373 (4)
C8—C13	1.445 (3)	C18—Cl1	1.737 (2)
C9—N1	1.354 (3)	C19—C20	1.384 (3)
C9—C10	1.492 (3)	C19—H19	0.9300
C10—C11	1.532 (3)	C20—H20	0.9300
C10—H10A	0.9700	N1—N2	1.385 (2)
			
C6—C1—C2	118.6 (3)	C10—C11—H11A	108.8
C6—C1—H1	120.7	C12—C11—H11B	108.8
C2—C1—H1	120.7	C10—C11—H11B	108.8
C3—C2—C1	121.3 (3)	H11A—C11—H11B	107.7
C3—C2—H2	119.4	C14—C12—C13	117.88 (19)
C1—C2—H2	119.4	C14—C12—C11	125.50 (19)
C2—C3—C4	119.7 (3)	C13—C12—C11	116.62 (18)
C2—C3—H3	120.1	O1—C13—C8	122.14 (19)
C4—C3—H3	120.1	O1—C13—C12	122.5 (2)
C3—C4—C5	120.0 (3)	C8—C13—C12	115.31 (18)
C3—C4—H4	120.0	C12—C14—C15	128.6 (2)
C5—C4—H4	120.0	C12—C14—H14	115.7
C6—C5—C4	119.8 (2)	C15—C14—H14	115.7
C6—C5—H5	120.1	C20—C15—C16	117.5 (2)
C4—C5—H5	120.1	C20—C15—C14	119.6 (2)
C5—C6—C1	120.5 (2)	C16—C15—C14	122.9 (2)
C5—C6—N1	120.5 (2)	C17—C16—C15	121.3 (2)
C1—C6—N1	119.0 (2)	C17—C16—H16	119.3
N2—C7—C8	112.04 (19)	C15—C16—H16	119.3
N2—C7—H7	124.0	C18—C17—C16	119.3 (2)
C8—C7—H7	124.0	C18—C17—H17	120.3
C9—C8—C7	104.84 (19)	C16—C17—H17	120.3
C9—C8—C13	123.08 (18)	C17—C18—C19	121.3 (2)
C7—C8—C13	132.08 (19)	C17—C18—Cl1	119.5 (2)
N1—C9—C8	106.97 (18)	C19—C18—Cl1	119.2 (2)
N1—C9—C10	129.34 (19)	C18—C19—C20	119.0 (2)
C8—C9—C10	123.66 (19)	C18—C19—H19	120.5
C9—C10—C11	108.14 (18)	C20—C19—H19	120.5
C9—C10—H10A	110.1	C19—C20—C15	121.4 (2)
C11—C10—H10A	110.1	C19—C20—H20	119.3
C9—C10—H10B	110.1	C15—C20—H20	119.3
C11—C10—H10B	110.1	C9—N1—N2	111.14 (18)
H10A—C10—H10B	108.4	C9—N1—C6	129.95 (17)
C12—C11—C10	113.84 (19)	N2—N1—C6	118.87 (17)
C12—C11—H11A	108.8	C7—N2—N1	105.00 (17)
			
C6—C1—C2—C3	0.3 (5)	C11—C12—C13—C8	−15.1 (3)
C1—C2—C3—C4	0.0 (6)	C13—C12—C14—C15	−179.6 (2)
C2—C3—C4—C5	−0.6 (5)	C11—C12—C14—C15	−0.8 (4)
C3—C4—C5—C6	0.8 (4)	C12—C14—C15—C20	137.4 (3)
C4—C5—C6—C1	−0.4 (4)	C12—C14—C15—C16	−43.2 (4)
C4—C5—C6—N1	−178.9 (2)	C20—C15—C16—C17	−1.6 (3)
C2—C1—C6—C5	−0.1 (4)	C14—C15—C16—C17	179.0 (2)
C2—C1—C6—N1	178.3 (3)	C15—C16—C17—C18	−0.3 (4)
N2—C7—C8—C9	0.0 (3)	C16—C17—C18—C19	1.3 (4)
N2—C7—C8—C13	−179.9 (2)	C16—C17—C18—Cl1	−178.43 (19)
C7—C8—C9—N1	0.5 (2)	C17—C18—C19—C20	−0.3 (4)
C13—C8—C9—N1	−179.6 (2)	Cl1—C18—C19—C20	179.4 (2)
C7—C8—C9—C10	−177.5 (2)	C18—C19—C20—C15	−1.7 (4)
C13—C8—C9—C10	2.3 (3)	C16—C15—C20—C19	2.7 (4)
N1—C9—C10—C11	−150.9 (2)	C14—C15—C20—C19	−177.9 (2)
C8—C9—C10—C11	26.7 (3)	C8—C9—N1—N2	−0.9 (2)
C9—C10—C11—C12	−48.7 (3)	C10—C9—N1—N2	177.0 (2)
C10—C11—C12—C14	−133.4 (2)	C8—C9—N1—C6	176.8 (2)
C10—C11—C12—C13	45.5 (3)	C10—C9—N1—C6	−5.3 (4)
C9—C8—C13—O1	169.9 (2)	C5—C6—N1—C9	−37.2 (4)
C7—C8—C13—O1	−10.2 (4)	C1—C6—N1—C9	144.3 (3)
C9—C8—C13—C12	−9.1 (3)	C5—C6—N1—N2	140.4 (2)
C7—C8—C13—C12	170.7 (2)	C1—C6—N1—N2	−38.1 (3)
C14—C12—C13—O1	−15.3 (3)	C8—C7—N2—N1	−0.5 (3)
C11—C12—C13—O1	165.8 (2)	C9—N1—N2—C7	0.9 (3)
C14—C12—C13—C8	163.8 (2)	C6—N1—N2—C7	−177.1 (2)

### Hydrogen-bond geometry (Å, º)

**Table:**

*D*—H···*A*	*D*—H	H···*A*	*D*···*A*	*D*—H···*A*
C14—H14···O1	0.93	2.43	2.804 (3)	104
C5—H5···O1^i^	0.93	2.53	3.320 (3)	143
C7—H7···O1^ii^	0.93	2.59	3.493 (3)	163
C17—H17···O1^iii^	0.93	2.40	3.260 (3)	154
C2—H2···Cl1^iv^	0.93	2.90	3.633 (3)	137

Symmetry codes: (i) −*x*+1, −*y*+1, −*z*+1; (ii) −*x*+1, *y*, −*z*+1/2; (iii) *x*, −*y*+2, *z*+1/2; (iv) *x*−1/2, *y*−1/2, *z*−1.
